# Investigating Disaster Response Capability and Exploring Solutions to Improvement Response Process of Hospitals to the Infectious Diseases Pandemic: An Explanatory Sequential Mixed-Methods Study

**DOI:** 10.4314/ejhs.v34i6.4

**Published:** 2024-11

**Authors:** Hojjat Farahmandnia, Asma Abdollahyar, Asiye Aminafshar, Mohammadmahdi Doustmohammadi, Sahar Salahi

**Affiliations:** 1 Health in Disasters and Emergencies Research Center, Institute for Futures Studies in Health, Kerman University of Medical Sciences, Kerman, Iran; 2 Department of Nursing, Zarand Branch, Kerman Islamic Azad University, Kerman, Iran; 3 Health Promotion Research Center, Zahedan University of Medical Sciences, Zahedan, Iran; 4 Department of Health in Disaster and Emergencies, Faculty of Public Health, Shahid Sadoughi University of Medical Sciences, Yazd, Iran; 5 Department of Nursing, Yasuj Branch, Yasuj Islamic Azad University, Yasuj, Iran

**Keywords:** disaster, response, capability, hospital, pandemic

## Abstract

**Background:**

Hospitals play a crucial role in local and national responses to various emergencies and disasters, including outbreaks of communicable diseases. This study aimed to investigate the disaster response capabilities of hospitals and to explore solutions for improving their response processes to infectious disease pandemics (IDPs) in Iran.

**Methods:**

This study employed an explanatory sequential mixed-methods design (quantitative and qualitative). In the quantitative phase, we assessed the response levels of reference hospitals (government, military, and private) in Kerman City, Iran, in 2023. This assessment utilized a researcher-designed questionnaire to evaluate the hospitals' capacity to admit pandemic patients. In the qualitative phase, we explored solutions to enhance the response processes of hospitals to IDPs through semi-structured interviews and a conventional qualitative content analysis approach.

**Results:**

The quantitative results indicated that the overall response level of hospitals to IDPs, with a mean score of 152.88±18.28, was moderate. The qualitative findings identified four main categories and nine subcategories of solutions to improve hospitals± response processes to IDPs.

**Conclusion:**

Hospitals are vital in providing quality healthcare during emergencies. Lessons learned from our institution's response to the COVID-19 pandemic will guide more efficient services and preparedness for future emergencies. Given the novel nature of the COVID-19 virus and the potential for similar pandemics in the future, a structured framework is needed to manage epidemic disasters effectively.

## Introduction

During disasters, hospitals are key components of the health and treatment landscape (1). They are expected to provide a safe environment for patients, visitors, and staff, while also delivering care to disaster survivors (2,3). Hospitals and other healthcare facilities play a critical role in both local and national disaster responses, particularly during outbreaks of communicable diseases (4). Their services encompass three main areas: community health, therapeutic treatments, and preventive care. In the context of infectious diseases, hospitals serve as the frontline defense for identifying potential outbreaks of emerging or reemerging diseases, with medical personnel in both high- and low-income countries tasked with recognizing and reporting emergencies (5).

A systematic review identified seven essential components for hospital readiness in response to biological events, including the SARS and influenza pandemics. These components include planning, surge capacity, communication, training and education, medical management, surveillance, and standard operating procedures. The establishment of an Africa Task Force for Coronavirus Preparedness and Response outlined six primary responsibilities, half of which pertained to hospitals: risk communication, supply chain management, surveillance (including screening at ports of entry), laboratory diagnosis, infection prevention and control, and clinical treatment of severe COVID-19 cases (6,7). Prolonged outbreaks can lead to the widespread transmission of diseases and a rapid increase in healthcare demands, placing significant strain on hospitals and the healthcare system (8-10). Under normal circumstances, hospitals often operate at full capacity, so even a minor increase in patient volume can create immense pressure and threaten their operational stability. To enhance preparedness for outbreaks, pandemics, or emergencies, hospital managers must prioritize appropriate actions (11-13).

COVID-19 spreads through direct contact with respiratory droplets and contaminated surfaces, with healthcare workers facing a heightened risk of infection in various clinical settings. Throughout the COVID-19 outbreak, many healthcare professionals worldwide contracted the virus, highlighting the need to address challenges faced by hospitals as they manage critically ill patients amid high workloads and limited staffing (14-17). Identifying these challenges can inform disaster management planning for the future. To improve operational capacity and standards, as well as to mitigate the catastrophic impacts of disasters, it is essential to recognize the capabilities, constraints, and shortcomings in hospital service provision. Evaluating the experiences of those in charge during disasters is one effective method to assess the strengths and weaknesses of disaster management programs (2). Although hospital-associated infection outbreaks constitute a small fraction of healthcare-associated infections, their common origin is transmission within healthcare settings, making timely identification critical for effective response and investigation. Current detection methods primarily rely on temporal or spatial clustering of specific diseases, often involving case counting and subjective assessment (18).

Zehang et al. examined hospital responses to the COVID-19 outbreak, noting that while challenging for healthcare professionals, a robust response framework enables hospitals to maintain operations for all patients without intra-hospital COVID-19 infections (19). Gupta et al. reported that key stakeholders in hospital-wide responses to COVID-19 included hospital administration, chiefs of staff, department heads, nursing leadership, and infection prevention teams (20). Macron et al. studied the response of Schiavonia Hospital, highlighting numerous challenges, including the need for adequate health emergency responses and logistical support to ensure the safety of healthcare workers (21).

The assessment of Iranian hospitals' response capabilities to IDPs, particularly in Kerman province, has received limited attention. This study aims to evaluate these capabilities and explore potential solutions for enhancing hospital response processes to IDPs in southeastern Iran. The preparedness and responsiveness of hospitals, as frontline defenders against pandemics, are crucial in public health emergencies. The findings of this study may inform effective decision-making for hospital officials and health services managers, helping to analyze governance structures, processes, strategies, and procedures, ultimately facilitating a quick recognition of existing hospital capacities and vulnerabilities in responding to IDPs.

## Methods

**Design:** This study was an explanatory sequential mixed-methods study (quantitative-qualitative).

**Study population:** The study's statistical population in the quantitative study consisted of nine reference hospitals for admitting infection patients, including governmental hospitals (H1-H3), military hospitals (H4-H6), and private hospitals (H7-H9) in Iran, Kerman Province, which was selected using a non-probability sampling method. To keep the information confidential, we assigned a letter to each hospital. The study's statistical population in the qualitative study consisted of 15 hospital managers who were interviewed in Iran, Kerman City, which was selected using purposive sampling. At least one year of professional experience as a hospital manager and a willingness to engage in the study were required for inclusion criteria. Lack of consent for study participation was the exclusion criterion. Purposive sampling was used, and between 45 and 55 minutes were allotted for the interviews.

**Data collection:** In the quantitative study, for data collection, the research team initially developed a questionnaire in two parts after an extensive review of the relevant literature to achieve good content validity. The first part included questions about the demographic characteristics of hospitals (Table 1). The second part contained 126 questions to investigate the response capability of (governmental, military, and private) hospitals to the IDP. Each question is in the form of a three-choice spectrum (not done = zero scores, incompletely done = score 1, and completely done = score 2). Scores between 0-84 were considered poor response levels, scores between 85-168 were moderate response levels, and scores between 169-252 were considered good response levels. The content validity of the questionnaire was approved by 10 professors of the Kerman University of Medical Sciences (including a psychiatrist, social health nurse, psychological health nurse, and social medical expert). The scale reliability was determined using a pilot study on 10 hospitals. Cronbach's alpha coefficient for the whole scale was 0.86.

**Table 1:** Demographic characterization of participating hospitals for administering COVID-19 patients

Hospital	H1	H2	H3	H4	H5	H6	H7	H8	H9
**Administrative Status**	Governmental	Governmental	Governmental	Military	Military	Military	Private	Private	**Private**
**Beds**	256	250	169	496	31	100	300	150	**162**
**Annual Occupied**	90%	85%	54%	90%	60%	78%	90%	70%	**52%**
**Bed/Year**									
**ICU Beds for Adults**	20 12(Covid -19)	98(Covid-19)	8 12(Covid-19)	30 35(Covid19)	4 (Covid-19)	10 (Covid-19)	20 (Covid-19) 6h.I.C. U	10(Covid-19) 6h.I.C. U	**8(Covid-19) 6h, IC. U**
**ICU Beds for**	0	0	0	3	0	0	11	3	**11**
**Pediatrics**									
**ICU Beds for**	0	0	0	0	0	0	4	0	**0**
**Neonatology**									
**Emergency Beds**	30	4(Covid-19)	12	66 Adult 32 Pediatric	12	24	48	14	**6**
**Emergency**	4	0	4	4	0	0	4	0	**0**
**Isolated Beds**									
**Microbiology**	Yes	Yes	No	Yes	Yes	Yes	Yes	Yes	**Yes**
**Laboratory**									
**Beds In Negative-**	6	1	0	6	0	0	2	0	**0**
**Pressure Rooms**									
**Number of**	150	120	100	150	0	0	0	0	**0**
**Antibiograms/Month**									
**Beds For Covid-19**	54	27	96	240	17	20	51	10	**3**

**Beds For Pregnant Women**	**0**	**0**	**0**	**5**	**0**	**17**	**29**	**9**	**40**

In the qualitative study, we explored solutions to improve the response process of hospitals to the IDP through conventional qualitative content analysis, and data were collected utilizing in-depth, semi-structured interviews, starting with open questions and progressively moving on to more in-depth ones. Please detail your experiences with the IDP against patients once the interviews got underway. What strategies do you suggest to improve the hospital's response process to the IDP? The questions were additionally tailored to each participant's observations on the IDP effects on hospital managers.

The researcher examines the recorded words multiple times during this inductive procedure to ensure complete comprehension. Then, the meaning-containing units (words, sentences, or paragraphs) that addressed the problems and difficulties encountered by the nurses in controlling the IDP were reduced and assigned a code. Similar codes that indicated related ideas were grouped into subcategories before being assigned to a category (manifest level). Each category was formed from a collection of content that had something in common, making the categories both internally and externally homogeneous. As a manifestation of the hidden meaning, the relationship between the underlying meanings in categories emerged as the central theme. The coding was managed by the MAXQDA 16 software trial version. This study employed strategies recommended by Lincoln and Guba for the reliability and validity of qualitative data (22).

**Data analysis:** In the quantitative study, we analyzed the extracted data in Microsoft Excel 2013. In the qualitative stage data gathered were analyzed by the content analysis approach proposed by Graneheim and Lundman (23).

**Ethical clearance:** The Ethics Committee of Kerman University of Medical Sciences approved this study. A cross-sectional design was employed in 2023, the code of number pajoohan is 99000549 and The Ethic approval Code is IR.KMU.REC.1399.464.

## Results

In the quantitative study, we evaluated nine selected hospitals that provided services to patients with infectious disease pandemics (IDPs). The specifications of these hospitals are detailed in Table 1.

Hospital number one served as the primary referral center for IDPs. The study results indicated that the overall response level of hospitals to IDPs, with an average score of 152.8±18.28, was at a moderate level. This suggests that to achieve an ideal response level, hospitals in Kerman require comprehensive planning in the response dimension of the disaster risk management (DRM) process, as shown in Table 2.

**Table 2:** The mean score of response performance of hospitals in southeastern Iran to the COVID-19 pandemic based on each hospital in each domain

Performances	D 1	D 2	D 3	D 4	D 5	D 6	D 7	D 8	D 9	D 10	D 11	D 12	D 13	D 14	Total Score
**H1**	10	16	23	26	11	7	17	14	20	13	8	20	6	10	**201.00**
**H2**	10	15	17	21	8	4	14	15	19	11	13	13	7	12	**179.00**
**H3**	9	14	17	22	11	6	6	9	9	5	8	14	8	15	**153.00**
**H4**	6	8	19	14	4	5	12	5	10	9	9	9	7	9	**126.00**
**H5**	8	15	6	20	8	7	15	14	19	13	10	8	9	9	**161.00**
**H6**	9	12	17	22	10	6	6	14	16	12	8	10	5	8	**155.00**
**H7**	9	12	19	12	5	4	9	8	17	10	11	11	3	10	**140.00**
**H8**	8	11	8	10	8	7	14	9	8	5	4	7	2	11	**112.00**
**H9**	9	14	9	16	9	4	8	8	18	11	12	20	4	7	**149.00**
**Total Mean Score**	**152.88 ±18.28**														

The findings revealed that government hospitals had a higher average response level to IDPs (177.66) compared to military hospitals (147.33) and private hospitals (133.66), as illustrated in Table 2.

**Figure 1:**
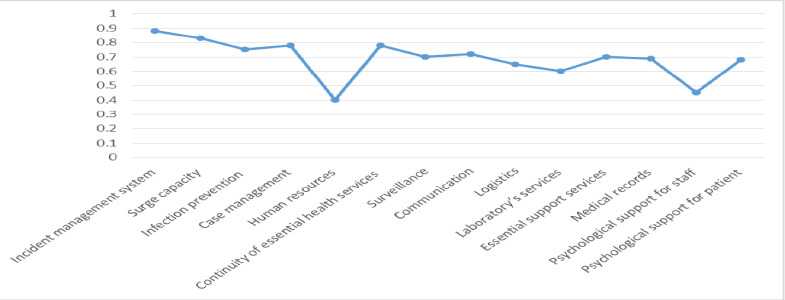
Minimum score of key components of the researcher-made questionnaire in the COVID-19 pandemic in participating hospitals

Additionally, the mean response rate of the hospitals involved in the study was high in terms of incident management systems and surge capacity. However, the response rate was low concerning human resources and psychological support for staff, as depicted in Figure 1.

The qualitative results identified four main categories and nine subcategories of solutions to enhance the hospital response processes to IDPs. These categories included: A) the necessity of conducting exercises in similar environments (simulating potential pandemics to prepare staff), B) educating all personnel involved in IDP services (preparing non-specialist workers for pandemics, training both clinical and non-clinical staff), C) effective management and leadership (appropriate policy-making and planning), and D) refining processes and structures (improving service delivery and ensuring the availability of protective equipment), as detailed in Table 3.

**Table 3:** Explore experiences and challenges of hospital managers' preparedness for timing response to the COVID-19 pandemic

Main categories	sub-categories
The necessity of holding exercises in the same environment	Performing exercises to prepare for possible Pandemics Experiencing scenes similar to real Pandemics Creating the experience of similar environments for the preparation of hospital Managers
Educating all members involved in providing services in the covid-19 pandemic	Preparation of non-specialist workers for Pandemics Training all members of the treatment and non-treatment team
Management and leadership	appropriate policy making suitable planning
Processes and structures	Process-related for providing services providing protective equipment

## Discussion

This study aimed to assess the response capabilities of hospitals to IDPs in southeastern Iran. The strategies employed by hospitals to confront IDPs are critical for effective pandemic control and management. The overall response rate of the participating hospitals was found to be at a moderate level, consistent with previous studies (24). For example, Zhang et al. reported that by implementing a structured response framework, hospitals in Shanghai were able to maintain operations for all patients without intra-hospital COVID-19 infections (19). Similarly, Chopra et al. highlighted essential factors for American hospitals during the COVID-19 pandemic, including the need for a full-time emergency manager and an operational task force (25).

In Iran, hospitals face significant economic challenges that hinder their ability to adapt and equip themselves adequately. To improve responses to IDPs, it is crucial to identify each hospital's capabilities, limitations, and weaknesses. The results showed that government hospitals had a higher response level compared to military and private hospitals. The Ministry of Health, Treatment, and Medical Education held primary responsibility for managing IDPs, which allowed governmental hospitals access to necessary resources. Consequently, private and military hospitals also played a role in admitting and treating infectious patients, despite having fewer facilities.

The study found that hospitals demonstrated a high mean response rate in terms of incident management systems. This aligns with findings from other studies (26), indicating that a well-structured management system can significantly improve risk management during biological events like the COVID-19 pandemic. An efficient management system minimizes negative incident impacts through clear task descriptions and transparent reporting channels.

Moreover, the hospitals assessed exhibited a high mean response rate concerning surge capacity. This dimension is vital, as hospitals typically operate at full capacity. It is advisable to utilize various hospital spaces, such as lobbies and waiting rooms, to accommodate an influx of patients (25). Surge capacity is a key aspect of hospital preparedness for emergencies and disasters (27). The ability to provide medical care during sudden increases in patient numbers is a primary concern for hospitals (28), and our study indicated that the surge capacity index was acceptable, particularly at the main referral center.

Conversely, the study revealed a low mean response rate concerning human resources, highlighting one of the greatest challenges in responding to IDPs. These findings are consistent with previous research (29). A shortage of clinical and non-clinical staff severely affects response capabilities. The nurse-to-patient ratio was reported at four to five patients per nurse, and during critical situations, this ratio could escalate to 1:1. In specialized wards, the ratio was approximately 2:1, while the physician-to-patient ratio ranged from five to ten patients per physician (30).

To address the human resource shortages, public hospitals have employed clinical students and utilized faculty from medical and nursing schools to support their efforts. However, recruitment of non-medical staff through temporary contracts has been ineffective due to the peak in COVID-19 cases in Kerman province. In contrast, private and military hospitals managed their medical and non-medical staffing more effectively, benefiting from sufficient financial resources.

Our study also found that hospitals achieved a high mean response rate regarding psychological and social support for staff, corroborating previous findings (31). Providing psychological support resources helps nurses cope with daily emergencies and enhances their preparedness. Targeted interventions, including training and psychological support, can bolster resilience and improve performance under uncertain conditions.

This study sought to identify solutions for enhancing hospital response processes to the COVID-19 pandemic. Four categories and nine subcategories emerged from interviews with hospital managers. Notably, managers emphasized the necessity of conducting exercises in similar environments as a key solution. Conducting basic training on disaster management and simulations can significantly improve disaster preparedness among hospital staff (32). Farhadloo et al. (2018) found that simulation training effectively enhances nurses' preparedness for triage during emergencies (33).

Additionally, the importance of educating all staff involved in IDP services was highlighted. Continuous monitoring of knowledge levels and training on hospital policies and procedures are essential (34). Participant interviews revealed challenges related to ineffective hospital management, arbitrary interventions, and inadequate policymaking, suggesting a need for competency-based training for hospital leaders (35).

The need for a stable organizational framework is paramount, as effective management directly impacts service quality. Poor management can demotivate staff and complicate decision-making processes. Globally, hospitals are crucial in responding to IDPs (36,37).

In conclusion, the study indicated that the overall response level of hospitals to IDPs was moderate. To achieve an ideal response, comprehensive planning in the DRM process is essential. In addition to preparing for natural and man-made disasters, health policymakers must strategize for outbreaks of highly contagious diseases. Therefore, healthcare center managers, along with higher-level officials in universities and the Ministry of Health, must allocate sufficient budget and human resources to enhance logistics.

Hospitals play a critical role in providing accessible quality healthcare. Addressing resource limitations requires innovative solutions. The lessons learned during our institution's response to the COVID-19 pandemic will guide us in delivering more efficient services and preparing for future emergencies. Given the evolving nature of COVID-19, the potential for future pandemics, and the diverse experiences of hospital managers, there is a pressing need for a framework that ensures effective and efficient management of epidemic disasters.

This study is notable for its nationwide scope, capturing challenges from various regions. However, due to the widespread impact of COVID-19, some interviews were conducted digitally rather than in person.
